# Tire mode shape categorization using Zernike annular moment and machine learning classification

**DOI:** 10.1038/s41598-024-59548-9

**Published:** 2024-04-25

**Authors:** Sudharsan Parthasarathy, Junhyeon Seo, Rakesh K. Kapania

**Affiliations:** Kevin T. Crofton Department of Aerospace and Ocean Engineering, Virginia Polytechnic Institute and State University, Blacksburg, VA 24060 USA

**Keywords:** Engineering, Mechanical engineering

## Abstract

This research proposes a framework for categorizing the radial tire mode shapes using machine learning (ML) based classification and feature recognition algorithms, advancing the development of a digital twin for tire performance analysis. Tire mode shape categorization is required to identify modal features in a specific frequency range to maximize driving performance and secure safety. However, the mode categorization work requires a lot of manual effort to interpret modes. Therefore, this study suggests an ML-based classification tool to replace the conventional categorization process with two primary objectives: (1) create a database by categorizing the tire mode shapes based on the identified features and (2) develop an ML-based surrogate model to classify the tire mode shapes without manual effort. The feature map of the tire mode shape is built with the Zernike annular moment descriptor (ZAMD). The mode shapes are categorized using the correlation value derived by the modal assurance criteria (MAC) with all ZAMD values for each tire mode shape and subsequently creating the appropriate labels. The decision tree, random forests, and XGBoost, the representative supervised-learning algorithms for classification, are implemented for surrogate model development. The best-performed classifier can categorize the mode shapes without any manual effort with a high accuracy of 99.5%.

## Introduction

Over the last several decades, tire vibration has been the subject of active research aimed at enhancing tire performance in terms of driving quality, safety, and passenger comfort. The tire vibration analysis is one of the major research areas to identify the characteristics of noise, vibration, and harshness called NVH. Various experimental and numerical analyses have been introduced and widely applied to determine the vibrational characteristics of the radial tire, such as mode shape, acoustic cavity resonance, etc. Recent improvements in computational capability and development in numerical techniques have led to high-fidelity simulation-based research environments using the finite element method (FEM), considering the high non-linearity coming from the tire’s geometry, material, and structural component.

In the field of NVH research in the tire industry, classifying mode shapes through vibration analysis plays a critical role in assessing and enhancing tire performance. With the emergence and proliferation of electric vehicles and the consequent absence of internal combustion engines, the tire NVH has gained more importance. In the automotive industry, understanding the natural frequency mode shapes of radial tires in the 0-500 Hz range is important for improving (tuning) road-tire Structure-Borne Noise (SBN) performance. Modal density is extremely high in this range. Efficiently characterizing and categorizing these modes based on the observed vibrational behaviors will help at the design stage and will be further useful for understanding, communicating, and comparing the vibrational behavior. It would save industry analysts time identifying modes and comparing modes between measured and predicted datasets, between two designs, and between different geometries. In general, it is necessary to repeatedly analyze the mode shapes and natural frequencies for newly designed tires to detect a specific mode that can degrade a tire’s performance in certain frequency ranges. For instance, the first circumferential mode can cause a slip issue, leading to the control degradation of the braking system. Also, the first radial mode can degrade the ride comfort, and higher-order radial modes can generate a high-level noise^1^.

In this study, we introduce a novel and streamlined approach to the vibration analysis of tires by facilitating the categorization of tire mode shapes using an advanced machine learning-based classification algorithm. Our primary objective is to enable users to efficiently identify and select mode shapes of interest by simply inputting the tire mode shape into our system. This approach minimizes the efforts required for conventional analysis by automatically interpreting specific mode shapes and eliminating those occurring within a predetermined frequency range.

Analytical attempts on tire vibration shapes began in earnest in the 1980s. Hunckler et al.^2^, had initially tried the FEM analysis to model the tire using shell elements for tire vibrational analysis. Kung et al.^3^ and Kung^4^, attempted to devise a process to classify the vibrational characteristics of the tire using modal features. They proposed multiple indices to describe the tire in-plane and out-of-plane mode shapes by counting the wave numbers in circumferential and meridional directions, individually and simultaneously. However, using a low-fidelity finite element model due to the low-level calculation capability in the 1980s, it took a lot of work to clearly distinguish the mode shapes where the waves of meridional and circumferential overlapped. In the late 1990s, Negrus et al.^5^, conducted a FEM analysis to derive the natural frequencies and mode shapes. They also categorized the tire mode shapes with the wave numbers along the circumferential direction. Wheeler et al.^6^, proposed a simplified method by only counting the tire waves along the meridional and circumferential directions. They used a relatively high-fidelity model and clearly provided categorized out-of-plane mode shapes for low-frequency ranges. However, this research also had limitations because the proposed indices could not categorize the in-plane mode shapes, including torsion and compression on the tire wall region. Patil et al.^7^, used Wheeler’s indexing approach to categorize the experimental results. Then, they showed the categorized tire mode shapes at relatively low frequencies achieved from the vibrational testing.

The previous representative research for classifying the mode shapes using indices had a common limitation: they required a manual effort to count the waves and classify the mode shapes along the meridional and circumferential directions (or the proposed directions). Classifying tire mode shapes using indices is practically difficult when the number of waves increases. Moreover, these conventional methods had the inconvenience of manually repeating the classification process when a structural configuration or boundary and load conditions are changed when developing a new design.

To overcome the identified limitation of previous studies, we propose a new framework using the advanced feature recognition (FR) method and a machine learning (ML) based classification model. Recently, ML-based approaches have been actively applied in the tire research domain to develop a ‘digital twin,’ which can replace the conventional calculation or analytical interpretation with the trained surrogate model. Zhu et al.^8^, developed the tire life prediction model based on image processing and ML algorithms. They built a worn tire image database and used them for training the *K*-nearest neighbor (*K*NN) classifier. Kuric et al.^9^, proposed an ML-based tire inspection system using the characteristic lines on the sidewall. They trained a convolution neural network (CNN) to discriminate the unusual patterns that can occur due to various defects. Lee et al.^10^, developed the ML-based model for tire pattern noise prediction in the early design stage. They successfully developed and validated their CNN-based surrogate model trained with the tread pattern images as input and pattern-induced noise database as the output. Wang et al. ^11^, suggested an FR framework to distinguish the mode shape of a circular plate using Zernike moment descriptor (ZMD) and modal assurance criteria (MAC). The ZMD is one of the FR methods that has the advantage of rotational invariance. ZMD is a set of Zernike polynomials (ZP) coefficients that can be used as the feature map by reconstructing the input data on a circular domain. MAC provides the correlation ratio between two modes by calculating the similarity of all components in the mode shape vectors. Wang et al.^11^, replaced the mode shape vectors with the ZMD for calculating the MAC to determine the similarities between the modes and to consider the rotational invariance of the circular plate vibration.

The development of the proposed ML-based tire classification model consisted of three steps: (1) modal displacement data processing, (2) label creation, and (3) classification tool development. Tire mode shape results from FEM analysis were transformed into a 2-D annular domain using a mapping process. The features of each mode shape were extracted using Zernike annular moments (ZAM)^12^ instead of Zernike moments^13^, as ZAM is capable of handling data on an annular domain, such as the projected half-section of a tire. The targeted characteristics of the input information were extracted by listing the constants of each Zernike annular polynomial (ZAP). Mode shapes were categorized by measuring the similarities of feature maps between each mode to generate category names (labels) using MAC. This process generated paired data, with the feature map as input and the categorized label as output.

To develop an ML-based categorization model, we utilized decision tree^14,15^, random forest^16^, and XGBoost^17^, which are representative supervised-learning classification algorithms. The derived mode-shape features were transformed into a new set of inputs capable of presenting the major characteristics through principal component analysis (PCA)^18^. PCA is a widely used method for reducing the dimensionality of input data in developing machine learning models. It is also applied in various conventional engineering problems, where it plays a significant role in handling complex systems and reducing their dimensionality^19–21^. The derived principal components (PCs) effectively delivered the key features of the original data by removing overlapping or unnecessary characteristics. The best-performing random forest successfully classified the mode shapes with an average accuracy of 99.5%.

The remaining part of this paper is organized into three sections after the introduction. “Data Generation” section explains the process of extracting and developing feature maps and labeled data using modal analysis results. “Method” section describes how to construct ML models that can be used to classify the tire modes. “Results and Discussion” section evaluates the performance of the developed classifier by applying the proposed framework in this study. The final section culminates this study and provides the conclusion.

## Data generation

### Tire FEM modal analysis

A typical tire is constructed with various layers made of different materials. The topmost layer of the tire is called the tread and is made of rubber to provide the required grip with the ground and cushion the outermost surface that is in direct contact with the road. In general, more rigid rubber is used in the side wall area to prevent large bending in the meridional direction. The belt is located under the tread, which is made of composite materials consisting of rubber and steel fiber, to provide the overall stiffness to the tire. Furthermore, the carcass, which is located under the belt, maintains the rigidity of the meridional direction. Additionally, the tire includes the body ply, a layer of fabric made from materials like nylon, polyester, or fiberglass embedded in rubber. This ply provides structural strength, maintaining the tire’s shape and supporting both the tread and the sidewall regions

**Figure 1:**
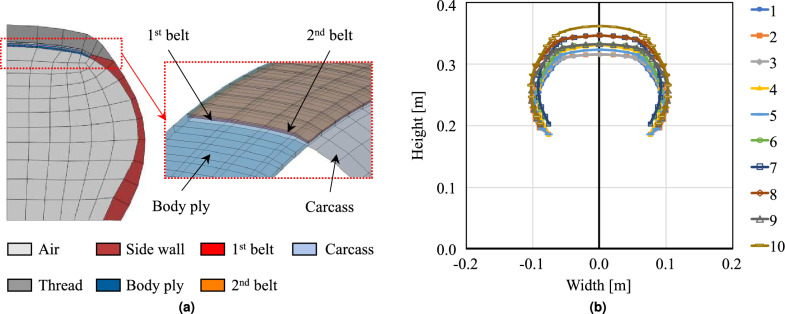
Tire FEM analysis model and geometries used for modal analysis and data generation, (**a**) Tire modeling configuration, (**b**) Configuration of ten different geometries.

The baseline finite element model used in this study is developed based on the Abaqus example model^22^. The planar model is developed with the element sets of air, carcass, rim, and rubber, which are major tire components as shown in Fig. 1a. This planar FE model is developed using an axisymmetric element to improve the calculation efficiency. The final model for the modal analysis is obtained by rotating and reflecting the original 2-D configuration about the rotation axis and symmetric plane. The material properties used for the tire model are as follows: (a) Rubber: Polynomial hyperelastic model, $E = 10^{6}$ N/m^2^, $\rho = 1100$ kg/m^3^, $\beta = 10^{-8}$, (b) Belt: Isotropic, $E = 172.2 10^{9}$ N/m^2^, $\rho = 5900$ kg/m^3^, $\nu = 0.3$, and (c) Carcass: Isotropic, $E =9.87 10^{9}$ N/m^2^, $\rho = 1500$ kg/m^3^, $\nu = 0.3$. Where *E* represents Young’s Modulus, $\rho$ is density, $\nu$ is Poisson’s ratio, and $\beta$ represents Raleigh damping factor proportional to stiffness. Further details of the baseline tire model are described in the reference^22^. The ten different tire models differ solely in geometry, while the material models for all tires remain the same.

The FEM modal analysis was conducted in multi-steps: (1) conduct static analysis for tire expansion using the planar model, (2) revolve and reflect model, (3) recalculate the static analysis for the full 3-D model using axisymmetric condition, (4) conduct the modal analysis to calculate natural frequencies and derive mode shapes. This research did not consider rolling and contact conditions that could cause other cavity effects.

The mode shape classification aims to categorize tire structures’ mode shapes, excluding the air configuration. In addition, the overall modal characteristics of a tire can be interpreted only by using the carcass rather than the whole tire model. Therefore, we used the modal displacements extracted from the carcass to develop FR data for each mode.

The modal displacements derived from FEM analysis can be extracted from Abaqus result files. Wang et al.^11^, suggested a new framework to recognize the mode shape features of a circular plate using the combination of FR and MAC. They used the ZMD, which describes regional features considering rotational invariance. Typically, the similarity between the two different modal displacements can be mathematically discriminated by using MAC, which calculates the correlation factor between two eigenvectors. In the case of axisymmetric domains such as disks and rings, the eigenvalue problem yields mode shapes with different phases at the same frequency. The conventional MAC differentiates such mode shapes, which is not desired. Therefore, Wang et al.^11^, developed the ZMD vectors for each mode, ignoring the rotational variance. Then, two ZMD vectors were used to compare the mode shapes.

This proposed method could classify the modal characteristics of the circular plate. This study referenced the framework proposed by Wang et al.^11^, but our method can be unique with comprehensive and interdisciplinary applications, including computational mechanics and advanced ML algorithms. Specifically, a feature map of vibrational characteristics is extracted using ZAMD, a modified concept of ZMD applicable to the data on the annular domain. These extracted modal features are expressed as vectors, which can be directly compared using MAC. The following sections describe the details of each step for data generation.

For data generation, we consider various tire sizes and shapes; ten different tire models were developed by modifying the outer diameter, wheel size, and width of the radial tire example model in Abaqus^22^ as shown in Fig. 1b. This study considered only free vibration, so the tread pattern is not considered, which can have a crucial effect on vibrational behavior for contact and loaded cases.

### Zernike annular moment for feature recognition

The current study focuses on ZAP^12^, which aids in finding geometrical moments of data in an annular domain. In general, a reconstruction method using geometrical moments (set of coefficients) and polynomials is one of the easiest ways to create a feature map for an image, and this set of coefficients can be used as a characteristic map of the input data. Derivation and mathematical details, along with the radial annular polynomials, are described in Mahajan^11,13^, which are studies about the ZAP.

Calculating the geometrical moment is one method by which descriptors can describe the characteristics of a figure. If continuous input data in a 2-D domain is expressed as *I*(*x*, *y*), we can write the geometrical moment, $m_{pq}$, in terms of polynomials in *x* and *y* as follows.

1 $$\begin{aligned} m_{pq}=\int _{-\infty }^{\infty } \int _{-\infty }^{\infty } x^p y^q I(x,y)dxdy \;\; p,q=0,1,2,... \end{aligned}$$

If we use the digitized image as an input and each pixel, having unit area, is described in terms of the *x* and *y* coordinates, the geometric moment of the input image is:

2 $$\begin{aligned} m_{pq}=\sum _{\bar{x}=1}^{N_x} \sum _{\bar{y}=1}^{N_y}\bar{x}^p \bar{y}^q I(\bar{x},\bar{y}) \end{aligned}$$

where $N_x$ and $N_y$ present the number of pixels along *x* and *y* directions, respectively. The coordinated $\bar{x}$ and $\bar{y}$ represent the discrete coordinates in digitized data within the function, $I(\bar{x}, \bar{y})$.

However, calculating the geometrical moment using a simple polynomial basis function, which is not orthogonal, requires high-order terms to reproduce complex geometry. Furthermore, a polynomial moment is highly sensitive to data having noise. To overcome these shortcomings, ZAM is used to process data on the annular domain for feature recognition. First, the set of moment weights for each order of ZAP is considered a feature of the input image. Then, we can build up the feature map as ZAMD composed of the feature list of ZAMs with respect to the ZAP orders.

This research used ZAP to reproduce the wave on the annular domain. If the input wave, $W(\rho ,\theta ,\epsilon )$, defines projections on the complex plane, the ZAM for each ZAP order *j* is:

3 $$\begin{aligned} ZAM_j=\frac{\langle W(\rho ,\theta ,\epsilon )\cdot ZAP_j \rangle }{\langle ZAP_j \cdot ZAP_j \rangle } \end{aligned}$$

where symbol $\langle \cdot \rangle$ represents function integration, i.e., $\langle f, g\rangle = \int \int f(a,b)g(a,b)dadb$. The value of ZAM for each ZAP order is calculated for the input wave, *W*, considering the Gram-Schmidt orthogonality condition, as follows^12^:

4 $$\begin{aligned} \begin{aligned} ZAM_j=&\frac{1}{\pi (1-\epsilon ^2)}\int _{\epsilon }^{1}\int _{0}^{2\pi }W(\rho ,\theta ,\epsilon )\cdot ZAP_j(\rho , \theta , \epsilon ) \rho d\rho d\theta \end{aligned} \end{aligned}$$

If the input data is described in the discretized $N_k$ number of normalized radii, $\rho _k$, and $N_l$ number of angles, $\theta _l$, then the moment of $ZAM_j$ value can be obtained as

5 $$\begin{aligned} ZAM_j=\frac{1}{\pi (1-\epsilon ^2)}\sum _{k=1}^{N_k}\sum _{l=1}^{N_l}I(\rho _k,\theta _l,\epsilon ) ZAP_j(\rho _k,\theta _l,\epsilon ) A_{kl} \end{aligned}$$

The input contour, wave (*W*), can then be reconstructed by recombining this characteristic map, *ZAM*, with weights for each *ZAP* (Eq. 6) term as follows:

6 $$\begin{aligned} W=\sum _{j=1}^{\infty }ZAM_j \cdot ZAP_j(\rho _k,\theta _l,\epsilon ) \end{aligned}$$

Therefore, the obtained set of $ZAM_j$ can be considered as a feature map for the input data.

### Modal displacement projection on annular domain

**Figure 2:**
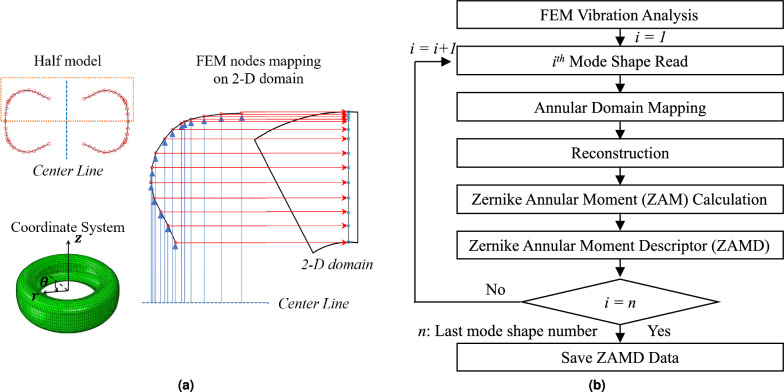
Modal displacement projection and overall data generation process, (**a**) Mapping process with the projected data using a quarter carcass, (**b**) Overall process of ZAMD data generation for each mode shape.

To extract features from the modal displacement of tires, we need to transform the data on the 3-D carcass to the 2-D domain through projection mapping. First, the modal displacement data on the specific location described in the $x-y$ coordinate system should be converted into the polar coordinate system expressed by radius (*r*) and azimuth angle $(\theta )$. In addition to calculating elemental area, the transformed modal displacement and polar coordinates for each element are also calculated by averaging the values at the nodes. The polar coordinates of the element and the elemental area are scaled so that it lies in the annular domain of inner radius $\epsilon$ and outer radius of one, as shown in Fig. 2a, where the FEM nodes are mapped onto a two-dimensional domain, enabling a detailed analysis of ZAMD in relation to variables *r* and $\theta$. ZAMD for each mode shape is calculated with the polar coordinates, elemental area, modal displacements (replacing *I* in Eq. 5), and ZAP. The overall process of ZAMD data generation is described in Fig. 2b.

**Figure 3:**
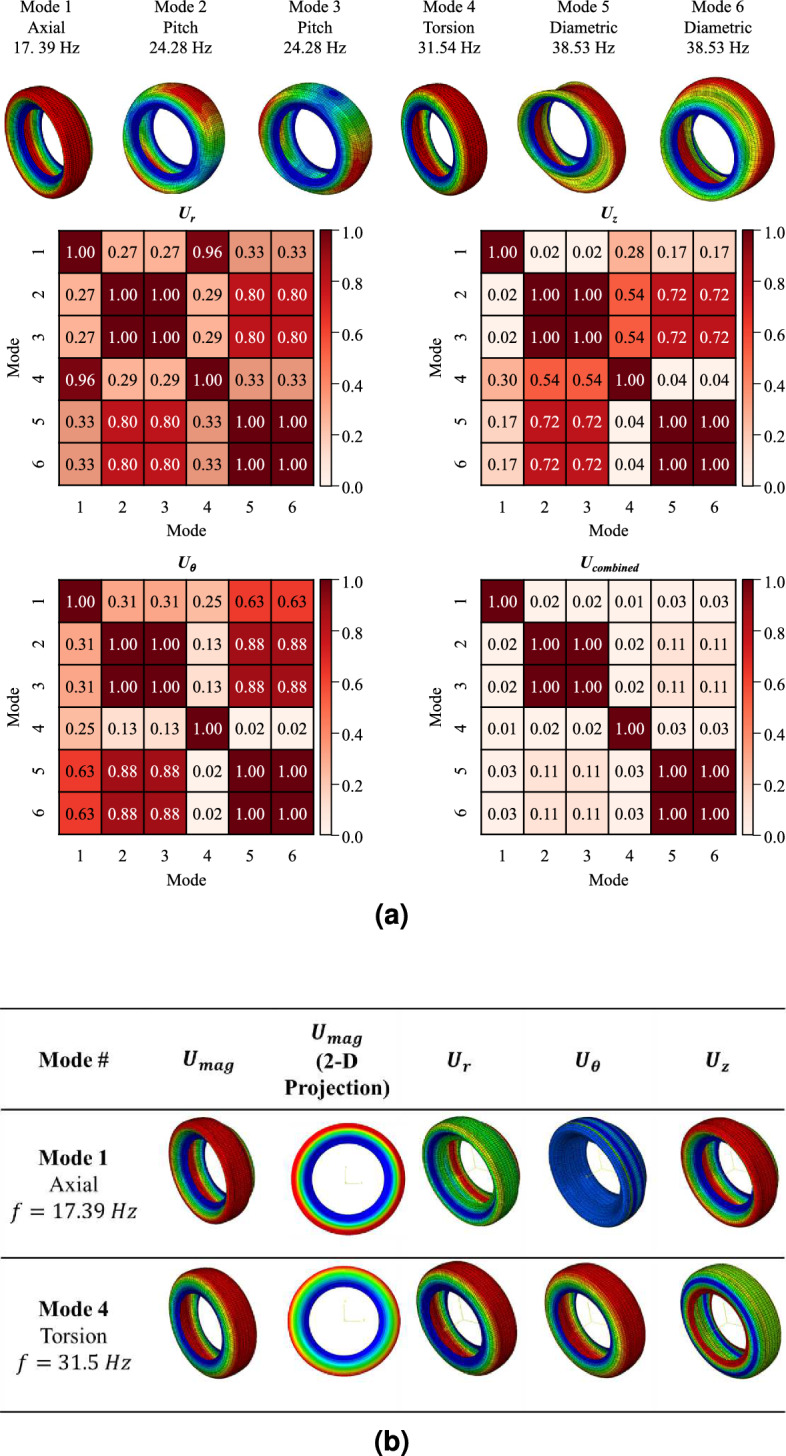
Advantages and importance of using $U_{comb}$ to create ZAMD database, (**a**) ZAMD-MAC table for the first six mode shapes using $U_{r}$, $U_\theta$, $U_{z}$ and $U_{comb}$, (**b**) Hoop direction displacement for axial and torsional mode shapes.

Even though we developed the feature maps for each mode shape using ZAMD, it is difficult to manually classify mode shapes by comparing the multiple ZAMD values. Therefore, in this study, the MAC is used to measure the similarity of mode shapes, calculating the correlation between each ZAMD vector by following the method proposed by Wang et al.^11^. The traditional MAC formulation is modified by replacing the eigenvectors with the ZAMD vector. Each ZAMD vector for a mode shape consists of Zernike annular moment amplitude $|Z_{n,m}|$, i.e., the contribution of each Zernike annular polynomial. The calculation of MAC for two ZAMD vectors for $i^{th}$ and $j^{th}$ mode shape, is as follows:

7 $$\begin{aligned} &ZAMD\text{- }MAC = \frac{\sum _{k=1}^{K} (|Z_{n,m}|_{i} - |\overline{Z}_{n,m}|)(|Z_{n,m}|_{j} - |\overline{Z}_{n,m}|)}{\sqrt{[\sum _{k=1}^{K} (|Z_{n,m}|_{i} - |\overline{Z}_{n,m}|)^2] [\sum _{k=1}^{K} (|Z_{n,m}|_{j} - |\overline{Z}_{n,m}|)^2]}} \end{aligned}$$

where *k* denotes the $k^{th}$ ZAM, i.e., each combination of *n* and *m* corresponds to a particular *k*. ‘*K*’ represents the maximum number of ZAM considered, and $|\overline{Z}_{n,m}|$ denotes the mean Zernike annular moment amplitude of all modes. When the MAC value is close to one, we can interpret that both vectors (mode shapes) have the same features and can be categorized as the same mode. Conversely, when this MAC value is close to zero, we can determine that the two targeted modes have no association and belong to different mode categories. Using the ZAMD-MAC approach proposed in this research, the classified results for the initial six mode shapes are described in Fig. 3a.

By only using modal displacement magnitude ($U_{mag}$), ZAMD-MAC cannot discriminate the axial and torsional modes. For axial and torsional modes, the modal displacement magnitudes equal zero at the rim with the pinned boundary condition and assume maximum values near the tire’s crown. These similar features make it difficult to categorize the mode shapes with specific vibrational characteristics such as torsion, in-plane shear, and high-bending modes at high frequencies. Therefore, it is necessary to consider additional rotational factors to distinguish axial, bending, and rotational motions, as shown in Fig. 3b.

We developed $$U_{comb}$$ by concatenating the modal displacement vectors $$(U_r, U_\theta$$, and $$U_z)$$ to build a ZAMD database by capturing the rotational features of mode shapes. The results of ZAMD-MAC are shown in Fig. 3a.

**Figure 4:**
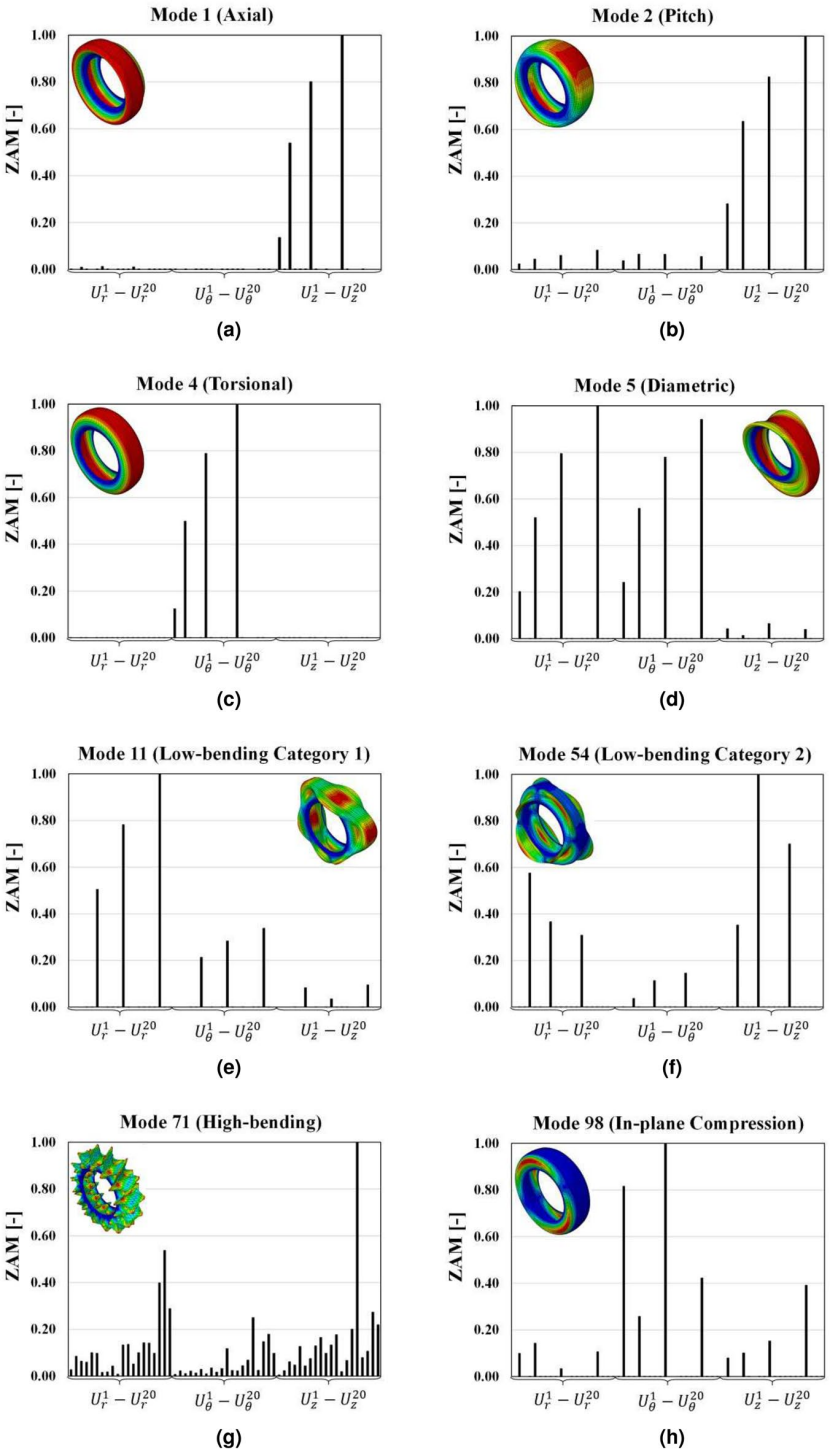
Representative mode shape categories and corresponding normalized ZAMD lists, (**a**) axial, (**b**) pitch, (**c**) torsion, (**d**) diametric, (**e**) low-bending radial or lateral (category 1), (**f**) low-bending non-radial or non-lateral (category 2), (**g**) high-bending, and (**h**) in-plane compression.

The eight representative mode shapes are applied to label the tire mode shapes as shown in Fig. 4. Finally, we can develop pairs of input and output data sets from the categorized mode shapes using the ZAMD-MAC.

If we have modal analysis results of a new radial tire model, we need to derive a ZAMD matrix for developing a new feature map. A ZAMD table consisting of the new mode shape results for the new design can be compared with an existing ZAMD, and MAC calculation can be used to classify the mode shapes of the new tire. However, as per the purpose of this research, we intend to replace this manual process with an ML-based classifier. The detailed method for creating such a classifier, based on the generated data, is described in the following section.

### Category labelling: representative tire modes

**Figure 5:**
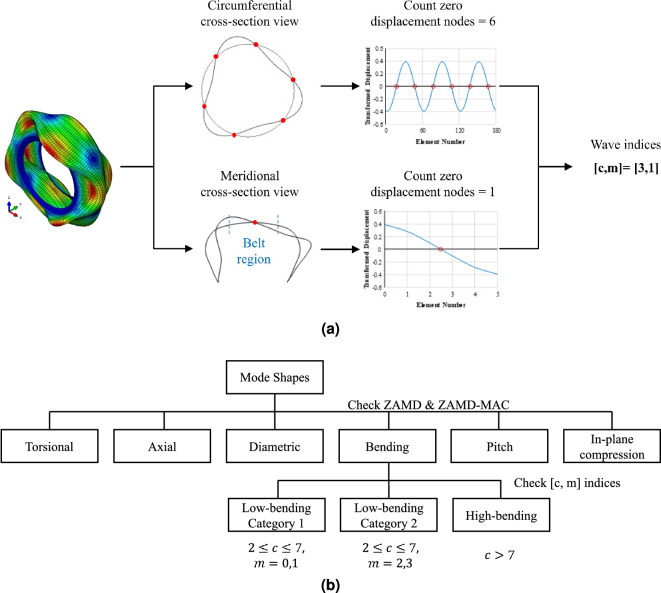
Obtaining [*c*, *m*] indices that aid in labeling bending mode shapes into three-categories, (**a**) Example process to determine [*c*, *m*] indices by flattening approach, (**b**) Labeling mode shapes using ZAMD and [*c*, *m*] indices.

The categories for the modes are chosen based on their dominant direction of modal displacement and effect on structure-borne noise, ride comfort, vehicle control, and damages to a specific system of a vehicle, i.e., braking system. A combination of ZAMD-MAC and two indices representing the wave features of tires are used to label the data by an automated process. The indices are in relation to the tire belt package, which is similar to a thin-walled cylinder, where ‘*c*’ represents the number of sinusoidal waves in the circumferential direction, while ‘*m*’ represents the number of waves in the meridional direction^6^. The [*c*, *m*] indices are obtained by the ‘flattening’ approach implemented by Kung et al.^3^. In this approach, the flattened carcass surface acts as the new neutral surface, and local mode shapes for each element are obtained using the local element coordinate system. The [*c*, *m*] indices are obtained for a low-bending category I mode shape in Fig. 5a, where the mode shape is exported along two planes to capture the waves along the circumferential and meridional direction.

The axial category includes modes with the modal displacement dominant in the axial direction, with either the belt region in rest or undergoing rigid body motion in the circumferential direction. In terms of the [*c*, *m*] indices, these modes will always have $c=0$. These modes impact the vehicle’s lateral control and occur at low, mid, and high-frequency ranges.

The torsional category includes modes with the entire tire undergoing shear or the sidewalls undergoing shear while the belt region remains stationary. These modes cause a slip or affect the vehicle’s braking system.

The pitch category includes modes where the belt package undergoes rigid rotation about the diametric axis^6^, an imaginary line passing through the center of a tire and dividing it into two halves. These modes have a significant impact during vehicle cornering. The modes in the diametric category include a rigid translation of the belt region about the diametric axis and impact the ride comfort of the vehicle.

The modes in the low-bending category 1 are of particular interest to automotive manufacturers as they significantly impact the ride comfort of the vehicle. The belt region for these modes undergoes bending in the circumferential direction, while in the meridional direction, they undergo rigid body translation or rotation. Regarding [*c*, *m*] indices, the ‘*c*’ value lies between 1 and 8, while the ‘*m*’ value is either 0 or 1. These modes generally occur at lower frequency ranges.

The structure-borne noise is caused due to two sets of categories of modes. The first is the low-bending category 2, where the ‘*c*’ value lies between 1 and 8, while the belt cross-section undergoes bending (*m* is 2 or 3). The second is high-bending category modes with a ‘*c*’ value greater than 7.

The final category is the in-plane compression modes, where the tire’s sidewalls undergo compression due to shear in the opposite direction while the belt region remains stationary. The number of full compression waves can range from 1 to 6 based on the tire stiffness and mass. The representative mode shapes for each category are shown in Fig. 4.

The labeling of mode shapes is a two-step process, as shown in Fig. 5b. ZAMD-MAC values and ZAMD vectors are used as the first filter to classify the mode shapes and label them into six broad categories, i.e., axial, torsional, pitch, diametric, in-plane compression, and bending. A constraint of $0.5\le ZAMD\text{- }MAC\le 1$ is ensured to group modes with similar shape features. To identify the grouped modes as axial, torsional, pitch, diametric or in-plane compression, we use the ZAMD values.

The second filter using [*c*, *m*] indices is meant to classify the modes that do not fall into the first classification filter, i.e., bending. The bending modes are further classified into low-bending category 1, low-bending category 2, and high-bending modes.

The [*c*, *m*] indices are not used for the entire classification process as these indices cannot clearly distinguish axial, torsional, and in-plane compression modes (all have $[c, m] = [0, 0]$). Similarly, ZAMD-MAC and ZAMD values can not be used to classify all the modes as it would lead to too many categories based on the shape features. ZAMD-MAC values would be $\ge 0.5$ for modes with the same *c*-index. This leads to two drawbacks: one is that there would be too many categories as the circumferential wave numbers range from 1 to 15 in the low to mid-frequency range. Although all high circumferential wave numbers mode ($c\ge 8$) contribute to noise, it is trivial to categorize them individually. The second drawback is that the motion along the meridional direction is equally important as it plays a vital role in vehicle control and structure-borne noise. Thus, a combination of ZAMD-MAC and [*c*, *m*] indices are used to classify the mode shapes into eight categories.

## Method

### Principal component analysis for input dimension reduction

When developing an ML-based classification or regression model, a large number of input features cause convergence issues and also decrease the accuracy due to the complexity of the surrogate model architecture. Therefore, it is necessary to reduce or optimize the number of data features used for input of the ML model. In particular, architecture simplification is a very efficient way to secure computational efficiency and prevent overfitting; it should be specifically used for an ML-based surrogate model that receives a large amount of information as input.

The PCA is a method for reducing input data dimensions for ML model development^18,23,24^. The PCA is a mathematical way to derive the PCs through singular value decomposition (SVD), which can represent data attributes by considering the relationships between each input feature. The PCA aims to minimize the loss of characteristics of existing data while reducing the dimension.

In relation to the current study, we build feature maps as the ZAMD vector using the modal displacements along radial $(U_r)$, rotational displacements along the hoop direction $(U_\theta )$, and axial displacements $(U_z)$. For each displacement, ‘*n*’ number of Zernike annular polynomials is used to derive the ZAM values. Therefore, a total of ‘3*n*’ number of ZAM values compose a ZAMD feature vector. However, all the information in the ZAMD vector is not considered significant for representing features of a mode shape. Each order of the ZAP can almost have the same weight, which can be considered meaningless when categorizing the target modes. PCA is applied to the ZAMD data, which helps in retaining important features and eliminating the least important ones. A more detailed explanation of the application PCA and related studies is presented in the later section.

### Classification algorithms

The choice of ML algorithms is highly dependent on the type of data; hence, it is important to try and compare different algorithms. Three such classification algorithms, namely decision tree, random forest, and XGBoost, are implemented and compared. Detailed information related to the architectures of each algorithm can be found in the references cited.

A decision tree is one of the supervised learning algorithms for classifying or regressing the output through a series of conditional statements^15^. Typically, functions such as purity of the leaf node or entropy functions are widely used as loss functions during training. In this study, the Gini (*g*) index^25^ was employed as the objective function. Additionally, ‘pruning’ is applied to mitigate the risk of overfitting^26^. The major limitations are a decrease in model efficiency due to large input features, overfitting issues for complicated problems that require more complex trees, and lower reliability. Random forest and XG boost algorithms include an advanced ensemble method applied to the decision tree algorithm to overcome the above-stated limitations.

An ensemble method is an advanced ML approach using multiple and independent trained models to increase accuracy and generalization capability^16^. A typical ensemble model combines multiple independent surrogate models into one to predict the label (classification) or estimate the values (regression). Various ensemble methods are presented, but typically, “bagging” and “boosting” are most widely applied for decision tree algorithms. Bagging^16^ is the short form for “bootstrap aggregation”, and this algorithm samples separate sets of data by allowing a replacement (bootstrapping) and combining (aggregated) them. These new data sets are used for training the individual decision trees and getting multiple estimations from them. The random forest is a popular ensemble model with multiple decision trees via bagging. Generally, bagging has the advantage of reducing the variance of predictions and avoiding overfitting.

Another famous ensemble method for tree algorithms is “boosting”^17^. A boosting algorithm trains the multiple trees in a sequential process, unlike a parallel training process in random forest development. During the sequential process, each decision tree considers a weak classifier, estimates the results, and calculates the loss (error). This loss is considered a residual and was used to develop the weighted dataset to enhance the effects of the data that failed to predict the correct outputs. The final output can be estimated from the ensembled results from individuals’ weighted predictions, and this final prediction is considered the strong classifier’s estimation. This boosting algorithm continuously improves the surrogate model’s accuracy through the consequent calculations, so the overall features are very similar to the neural network. There are various ways to update the residual and update models, such as adaptive boost (AdaBoost)^27^, gradient boost^28^, extreme gradient boost (XGBoost)^17^. More detailed theoretical backgrounds of each theory are described in the cited references. We employed the XGBoost package^29^ in Python, an advanced implementation of the gradient boosting framework. This approach augments the conventional gradient descent methodology intrinsic to standard gradient boosting algorithms, facilitating the identification of an optimal model with improved efficiency and predictive accuracy. Parameters such as learning rate ($lr = 0.01$) and the number of estimators ($n_{estimators} = 100$) were fixed for all the remaining parametric study-related XGBoost. The learning rate controls the contribution of each tree to the final prediction, while the number estimators represent the number of boosting rounds or trees in the ensemble. The PCA process and development of decision tree, random forest, and XGBoost were performed through Scikit-Learn^30^ and Pandas^31^, which are Python machine-learning packages.

## Results and discussion: example for tire mode classification

**Figure 6:**
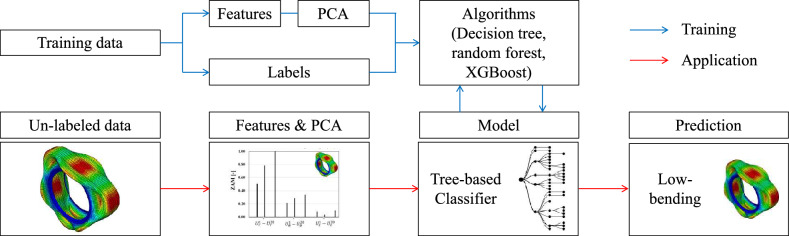
Overall process of training classifiers and their application to predict the category for un-labeled data.

This section presents a comprehensive flowchart outlining the steps involved in developing automated tire mode shape categorization tools. Figure 6 provides a visual representation of the overall training process, including the application of the trained classification model to unlabelled data or new tire designs for mode shape categorization.

To enhance the accuracy of the classification model, parametric studies were conducted for the decision tree, random forest, and XGBoost algorithms. Various parameter configurations were explored, and performance metrics such as accuracy, precision, and recall were used to evaluate and compare the models’ performance. By following this comprehensive flow and conducting parametric studies, we aimed to develop highly accurate and robust surrogate models for tire mode shape categorization, thereby improving tire design and performance evaluation processes.

The hardware details of the machine used for the current study are as follows: (a) Processor: Intel(R) Xeon(R) W-2265 CPU @ 3.50GHz 12 cores, and (b) RAM: 128 GB.

### ZAMD data generation

A total of 2,000 datasets are generated with the 200 mode shapes for the ten different geometries, as shown in Fig. 1a. For testing, we strategically allocated 20% of the total dataset, comprising 400 data points, specifically for the testing phase. This was done after ensuring a diverse representation from 200 mode shapes across 10 different tire geometries in the initial dataset of 2000 data points. Consequently, the remaining 1600 data points, representing a wide range of tire designs, were used for training. This approach aims to mitigate design-specific bias in our testing and training data, potentially contributing to the improved robustness and generalization capability of the model. The associated dataset consists of the ZAM vector as an input and the mode shape category label representing the features of the tire modal deformation. The dataset was used to train various tree-based surrogate models that can provide the category name without any additional process. Once this system is developed, the user can simply input the ZAM vector to determine the features of the target mode shape and immediately identify the mode category. We employed the decision tree and ensemble algorithms. Also, the original ZAM data and set of principal components are utilized for two independent sets of data to train the classification model to verify the effects of PCA. The original input data without any pre-processing includes 60 features coming from 20 weights of ZAM polynomials for the radial ($U_r$), hoop ($U_{\theta }$), and axial ($U_z$) modal displacements, respectively.

### Development of ML-based classifier

Parametric studies were conducted to derive the best-performed parameters for each algorithm application. First, we determined the decision tree architecture to identify the optimum parameters for single decision tree applications and further ensemble methods applications.

In the initial stage of the parametric study, we used a relatively large step to observe the optimum parameters. Then, we narrowed the specific ranges of each parameter using the finer resolution to obtain the final values. We repeated this process ten times and found the best-performed case to avoid biased results. The number of principal components ($n_{PC}$) and the depth of the decision tree (*d*) are observed for all models. Furthermore, the number of estimators ($n_{tree}$) is determined for both ensemble methods. Especially for the random forest, the number of features ($n_{feat}$) is also observed to develop the most efficient surrogate model. The prediction accuracy and relevant classifiers’ parameters are described in Table 1 for each algorithm.

**Table 1:** Accuracy difference for parametric study results using 400 testing data set.

$n_{PC}$	Decision tree		Random forest				XGBoost		
	*d*	Acc. [%]	*d*	$n_{feat}$	$n_{tree}$	Acc. [%]	*d*	$n_{tree}$	Acc. [%]
10	15	96.5	12	10	34	98.5	14	54	97.5
15	13	96.0	14	10	30	96.5	15	48	97.0
20	17	96.5	16	4	28	99.0	15	35	98.0
25	16	96.5	15	6	28	99.0	14	42	98.0
30	13	97.0	14	2	28	99.0	13	55	98.0
35	15	96.5	16	4	30	99.0	11	50	98.5
40	17	96.0	14	10	28	99.5	13	42	98.5
45	16	96.5	14	8	28	99.5	14	53	98.5
50	9	97.0	16	8	30	99.0	13	49	98.5

### Discussion

**Figure 7:**
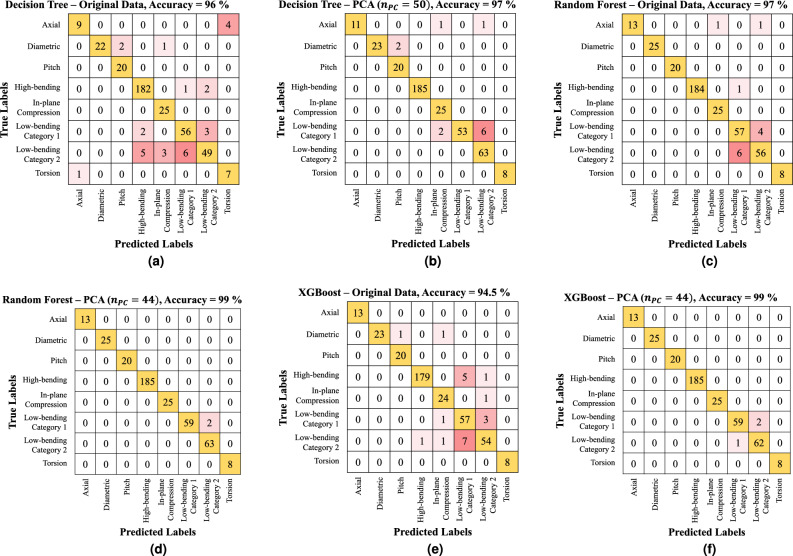
Prediction performance tables for (**a**) decision tree with original data, (**b**) decision tree with PCs, (**c**) random forest with original data, (**d**) random forest with PCs, (**e**) XGBoost with original data, (**f**) XGBoost with PCs.

Table 1 shows the sensitivity of the number of principal components and provides the optimum parameters for better predictions. If the number of principal components exceeded 20, all models predicted category labels with very high accuracy (> 97%). From Fig. 7 and Table 1, the application of PCA reduced the input dimension to improve the data efficiency and the accuracy of category prediction.

Figure 7 shows the prediction results using the testing data set for all model applications trained with the original data and a set of principal components. Each model’s performance accuracy improved when the classifiers were trained with the principal components. In particular, we found that the XGBoost model is most sensitive to whether we use the principal components or the original data set. The original data model has 94.5% accuracy compared to the principal component application, which has an accuracy of 99%.

Considering each model in detail, the single decision tree model predicted the label correctly with 97% accuracy. This high accuracy proves that the proposed ZAMD vector can represent each category’s features well.

For the ensemble methods, the random forest provided very accurate category predictions, as accuracy = 99.5%, when $n_{PC}$ = 32, $n_{tree}$= 28, *d*= 15, as shown in Fig. 7d). The best-performed XGBoost classifiers also correctly predict the labels with the accuracy of 99.3%, when $n_{PC}$ = 41, $n_{tree}$= 42, *d*=13, as shown in Fig. 7f).

The training and testing times for XGBoost, utilizing the best set of parameters and principal components, are 0.3890 seconds and 0.0060 seconds, respectively. In comparison, for Random Forest under similar circumstances, the training and testing times are 0.0588 seconds and 0.0030 seconds. The macro average of f1-score, precision, and recall for XGBoost, utilizing the best set of parameters and principal components, are 0.9939,0.9939, and 0.9940, respectively. In comparison, for Random Forest under similar circumstances, the f1-score, precision, and recall are 0.9960, 0.9962 and 0.9959.

Three low-bending cases were incorrectly categorized for the prediction failed cases for each category 1 and 2, regardless of whether the modes included the radial or lateral deformation behaviors. The potential reasons are likely attributable to similar shape features of certain modes in these categories. As previously discussed, only half of the tire model is considered for extracting the shape features due to two major reasons: firstly, this approach is computationally more efficient owing to the exploitation of symmetry; secondly, the usage of Zernike annular polynomials, which are radial and azimuthal functions, allows for an effective orthogonal projection of the half tire model onto an annular domain to facilitate the extraction of shape features. However, the potential drawback of considering the half-tire model is that the shape features in the meridional or cross-sectional direction cannot be extracted completely. This could lead to mode shapes in two categories with the same circumferential wave number but different meridional wave numbers having similar shape features.

There are 124 low-bending modes out of the total testing cases, and only three of them could not be predicted (error = 1.61%). Therefore, the developed ML-based classifiers can be considered highly accurate surrogate models using the proposed ZAMD vector as a feature map of each tire mode category. The proposed framework can be used to categorize the mode shapes for analyzing the tire’s vibrational behaviors for new or improved designs.

## Conclusion

This research introduced a novel framework for developing an ML-based classification tool to automatically categorize tire mode shapes, significantly improving efficiency in tire design. The optimum classifier, employing a random forest algorithm, achieved an accuracy of 99%, confirming that the trained surrogate model can reliably serve as a digital twin, replacing iterative analyses in conventional tire design procedures.

Our study’s main objective was to develop a conceptual initiative for a digital twin capable of automatically determining mode shape categories, focusing on the relatively simple mode shapes in the low-frequency range. Ten different tire baseline FE models were employed to generate data, and an ML-based model was developed to predict mode shape categories from feature maps using a decision tree algorithm. Advanced ensemble methods, random forest, and XGBoost improved performance, with accuracies of 99.5% and 98.3%, respectively.

The proposed framework demonstrates the effectiveness and performance of our ML-based method, with potential applications in automating and streamlining tire design processes. Future research can explore simplifying the classification process by using contour images and convolutional neural network-based classifiers and extending the approach to mode shapes at higher frequency ranges, loaded and rolling tires, and localized displacement modes.

## Data Availability

The data that support the findings of this study are available from CenTiRe but restrictions apply to the availability of these data, which were used under license for the current study, and so are not publicly available. Data are however available from the authors upon reasonable request and with permission of CenTiRe.
